# The role of the robotic technique in minimally invasive surgery in rectal cancer

**DOI:** 10.3332/ecancer.2013.357

**Published:** 2013-09-26

**Authors:** Paolo Pietro Bianchi, Fabrizio Luca, Wanda Petz, Manuela Valvo, Sabine Cenciarelli, Massimiliano Zuccaro, Roberto Biffi

**Affiliations:** 1 Unit of Minimally Invasive Surgery, European Institute of Oncology, 20141 Milano, Italy; 2 Unit of Integrated Abdominal Surgery, European Institute of Oncology, 20141 Milano, Italy; 3 Division of Abdomino-Pelvic and Minimally-Invasive Surgery, European Institute of Oncology, 20141 Milano, Italy

**Keywords:** rectal cancer, total mesorectal excision, laparoscopic surgery, robotic surgery

## Abstract

Laparoscopic rectal surgery is feasible, oncologically safe, and offers better short-term outcomes than traditional open procedures in terms of pain control, recovery of bowel function, length of hospital stay, and time until return to working activity. Nevertheless, laparoscopic techniques are not widely used in rectal surgery, mainly because they require a prolonged and demanding learning curve that is available only in high-volume and rectal cancer surgery centres experienced in minimally invasive surgery. Robotic surgery is a new technology that enables the surgeon to perform minimally invasive operations with better vision and more intuitive and precise control of the operating instruments, promising to overcome some of the technical difficulties associated with standard laparoscopy. The aim of this review is to summarise the current data on clinical and oncological outcomes of minimally invasive surgery in rectal cancer, focusing on robotic surgery, and providing original data from the authors’ centre.

## Introduction

The most important advance in rectal cancer surgery over the last 30 years was the development, affirmation, and standardisation of total mesorectal excision (TME) [1]. TME is associated with reduction in local recurrence rate to <10% and improved survival in some series [2]. TME aims to completely remove the rectum with its perirectal fat, at the same time maintaining the integrity of the mesorectal fascia and preserving the autonomic nerves.

The available literature on minimally invasive TME for rectal cancer, as illustrated by the findings in a recent meta-analysis of randomised control trials [3], demonstrates that laparoscopic TME is feasible and associated with better short-term outcomes than open surgery, while maintaining equivalent oncologic safety. Nevertheless, laparoscopic TME for rectal cancer is not widely practiced [4–7]. The main reasons for this are the prolonged and demanding learning curve, and the technically challenging nature of the operations themselves, related to the lack of adequate space within the pelvis and difficulty of manoeuvring the rigid and cumbersome laparoscopic instruments [8]. It has been estimated that over 50 operations [9] are required for an experienced colorectal surgeon to achieve proficiency with minimally invasive techniques and such experience can only be obtained at high-volume centres. One way of overcoming this limitation would be to make minimally invasive operations technically easier, thereby reducing learning time and increasing the attractiveness of minimally invasive approaches for young surgeons [10–12].

Robotic surgery is a sophisticated new technology that improves the traditional laparoscopic surgery in different ways: it has high-definition three-dimensional vision, it translates the surgeon’s hand movements into precise movements of the instruments inside the patient, the camera is held and moved by the first surgeon, and a fourth robotic arm is available as a fixed retractor. All these aspects increase surgeon comfort and reduce fatigue [13]. The aim of this paper is to review the available data on robotic surgery for rectal cancer in comparison with traditional laparoscopic technique.

## Laparoscopic surgery in rectal cancer

A minimally invasive approach to TME has been shown to be feasible and oncologically safe in several trials, the two largest being the CLASICC trial [14], which recruited colon and rectal cancer patients, and the COREAN [15] trial, which recruited rectal cancer patients only. There was some concern about the higher rate of circumferential resection margin (CRM) involvement among rectal cancer patients undergoing low anterior resection in the laparoscopic arm (12.4%) of the CLASICC trial compared with the open arm (6.3%). This was probably due to the greater technical difficulty associated with the laparoscopic approach for rectal cancer—as indicated by higher conversion rate in the rectal laparoscopic than colon laparoscopic subgroup (34% versus 25%). However, in this study, the difference in CRM involvement did not translate into a difference in five-year disease-free survival between laparoscopically assisted and open surgery [16].

Furthermore, in the CLASICC trial, morbidity and mortality rates were high among laparoscopic cases (colon and rectal cancer) converted to open surgery. Thirty-day morbidity was 29% in laparoscopic cases and 45% in converted cases; in-hospital mortality was 1% in laparoscopic cases and 9% in converted cases. Some of this extra morbidity could have been due to more advanced cancers requiring conversion, but the longer operating times, greater technical difficulty, and need for a wide open incision in the converted cases, almost certainly contributed to these findings. Conversion to open surgery can also result in greater local recurrence rate [17]. Finally, participation in the CLASICC trial required that involved surgeons had to have performed at least 20 previous laparoscopic colorectal resections; it is possible that many operators were still in the learning phase of their activity at the time when the CLASICC study started (Table 1).

The COREAN trial was designed to assess the safety and efficacy of laparoscopic surgery in comparison with open surgery for cT3N0-2 mid and low rectal cancer without distant metastasis after preoperative chemoradiation therapy. The seven surgeons who contributed to this trial had done a median of 75 previous laparoscopic colorectal resections, and the three hospitals involved were high-volume centres, with over 200 rectal cancers treated annually. The trial found no differences for CRM positivity or TME quality, but documented short-term advantages for laparoscopic surgery over open one. The conversion rate was 1.2% (2/170). The lack of cT4 cases, the lower mean body mass index of patients (24.1 kg/m^2^ versus CLASICC 26 kg/m²), and greater surgeon experience matured in high-volume centres, may all have contributed to low conversion rate and good oncologic results of the COREAN study (Table 1).

Notwithstanding these findings, laparoscopic rectal resection is still a challenging procedure, even in expert hands [18]. The recent guidelines of the European Association for Endoscopic Surgery recommend that it should be performed only by expert surgeons at high-volume centres and in selected patients (cT4 cases should be excluded); if these conditions are met, the laparoscopic approach can be an acceptable alternative treatment option to conventional open surgery [19], maintaining its well-established advantages (less pain, less blood loss, shorter hospital stay, and better quality of life in the early postsurgical period).

## Technical aspects of minimally invasive TME

Laparoscopic low rectal resection is second only to reversal of Hartmann’s procedure in terms of colorectal operation difficulty [8]. The main problems are the lack of space within the rigid pelvis and the small operating field, so that exposure of the lateral and anterior rectal wall in particular is hindered. Furthermore, the view with the laparoscopic camera is often obstructed by smoke from cautery instruments, and a good view of the operating field often depends on the skill of the camera assistant, who must be experienced, thoroughly familiar with all the steps of the procedure, and also have an excellent relationship with the operating surgeon, understanding where he/she wants to go and what he/she wants to see. The robotic technology seems to offer some advantages to overcome the above-mentioned technical difficulties of laparoscopic procedure.

The only robotic system so far available is the da Vinci Surgical System (Intuitive Surgical, Mountain View, California, United States). This consists of a console, some distance from the patient where the surgeon sits while operating, and the robotic cart with its three or four robotic arms, which is in close proximity to the patient. Various instruments, attached to the robotic arms, are inserted into the patient through small incisions. The console provides a magnified high-definition three-dimensional view of the operating field through a stereo-endoscope fitted to one of the robotic arms, whose visual field and angle of vision are adjustable.

**Figure 1.**
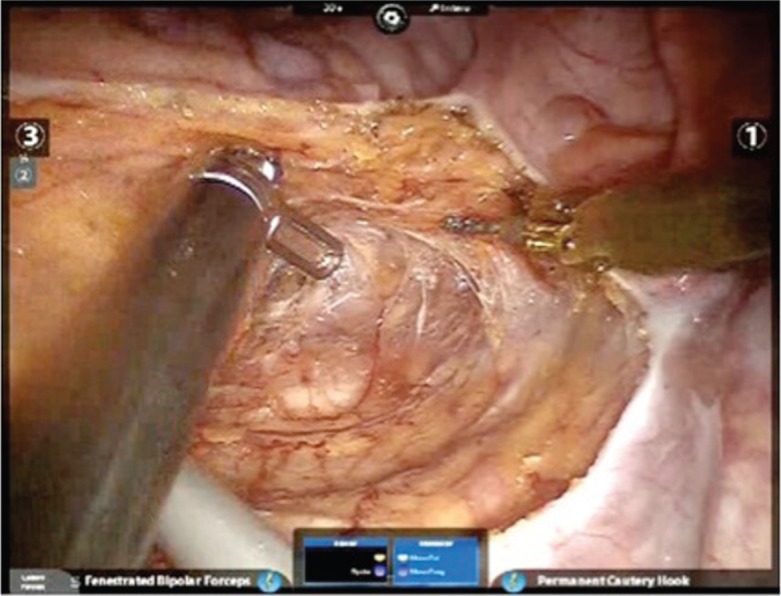
Robotic TME.

The surgeon manipulates two-finger joysticks (finger and wrist movements) at the console while watching the surgical field through the visual system. Movements of the surgeon’s hands are transmitted through the robot to the robotic arms, so that equivalent but scaled movements of the surgical instruments are made within the surgical field. The instruments have seven degrees of movement freedom and hand tremor is filtered out [13, 20]. The da Vinci system appears able to overcome many of the problems that characterise laparoscopic rectal resection. Initially, the three-dimensional high-definition camera is under the direct control of the surgeon, and the double optical system makes consistently clear vision more likely. Furthermore, the tremor-filtered, multiple-degree-of-movement instruments, which can be scaled relative to the surgeon’s hand movements, permit more precise dissection in the narrow space and render the more difficult steps of the operation easier. Tissue retraction with the robot is also easier, since the third arm can be used as a fixed retractor, and is always manipulated by surgeon (Figure 1) [21]. These potential advantages within the narrow space of the pelvis have been shown to be translated into concrete advantages for robotic radical prostatectomy [22], and in our opinion the excellent ergonomics and precise dissection offered by the robotic system can be helpful in colorectal surgery also for vessel ligation and flexure mobilisation. This is why we have standardised a ‘fully robotic’ technique for left colon and rectal resection [23].

## Literature review

Robotic TME has been introduced mainly in centres with a high volume of rectal cancer treated per year and by surgeons who are experts in minimally invasive surgery [24–38]. The published studies are small, not randomised, single centre series; despite the low evidence of data available, five meta-analyses [39–43] have already been published on this technique, indicating the marked interest in robotic surgery for rectal cancer. In the published series, the robotic technique is not standardised, rectal resection can be performed with a full robotic technique or using a hybrid procedure with some steps laparoscopy performed, but in all the studies, the TME step was performed robotically [24–28, 30–38]. Overall, the published studies are mainly retrospective or prospective single centre series, comparing mainly robotic rectal resection to laparoscopy, but recently some studies comparing robotic rectal resection with open surgery have also been published [44–46].

In comparison with standard laparoscopy, the most important finding for robotic rectal surgery is the lower rate of conversion to open surgery. In the meta-analysis in [39], robotic rectal resection was associated with a significant reduction of conversion rate in comparison with laparoscopy. In the meta-analysis in [40], conversion rates and estimated blood loss were significantly lower in robotic rectal cancer patients than in rectal cancer patient undergoing conventional laparoscopic resection (Table 2). The meta-analysis in [41] also reported a significantly lower conversion rate among robotic patients.

Conversion to open surgery is an important indicator of proficiency, generally being ~10–15% in laparoscopic rectal surgery, except for the COREAN trial, where the conversion rate was 1.1%. A preliminary publication of the COLOR II trial reported a 17% conversion rate to open surgery [47]. An RCT [robotic versus laparoscopic resection for rectal cancer (ROLARR)]—comparing laparoscopic with robotic rectal resection for cancer—is ongoing, and its first end-point is the conversion rate to open surgery [48].

Learning for minimally invasive surgery is a crucial issue, because laparoscopic colorectal surgery has a steep learning curve, particularly for rectal cancer, and robotic surgery seems to reduce the learning curve to 15/20 cases, as calculated in [49], or even avoided, as reported in [50].

In our Institute (European Institute of Oncology, Milano, Italy) three senior consultants approached the robotic technique in rectal surgery. One of them was highly experienced in laparoscopic surgery with >100 laparoscopic colorectal resections. In 90 cases of robot-assisted rectal resections performed by the expert laparoscopic team, the operative time did not change, as well as leakage (4%) and conversion rates (2%), thus confirming the data from the clinical series in [50]. The other two surgeons had a solid experience in open colorectal surgery but were less expert in laparoscopy; nevertheless, the learning curve was brief (ten cases for each surgeon) and the operative time reduced to <240 min in a short span of time [51]. Therefore, it is possible to assume that for an experienced colorectal surgeon the impressive facilitation of the performance of complex laparoscopic procedures offered by the robotic system does not imply to complete an advanced laparoscopy learning period. The overall series of the IEO group is presented in Table 3.

## Clinical and oncological outcomes

Looking at short-term clinical outcomes, robotic surgery did not show significant differences from laparoscopic technique in all the analyzed studies. Overall complications rate and length of hospital stay are similar for both techniques, and this can be easily explained because the operation performed robotically is the same than in laparoscopy, with the aid of a better technology. In comparison with open surgery, robot-assisted surgery maintains all the advantages of minimally invasive procedures [44–46] (Table 4).

Few data are available on sexual and urinary dysfunctions, which is a crucial issue to be evaluated, because laparoscopy did not add advantages in term of sexual/urinary dysfunctions preservation [52, 53]. Moreover, the MRC CLASICC trial raised a concern that laparoscopic TME may be associated with increased rates of male sexual dysfunction compared with those of conventional open TME [54]. Otherwise, in robotic prostate resections, a better preservation of genito/urinary functions compared with open or laparoscopic procedure has been demonstrated [22]. To date, only three studies—evaluating genitourinary function after robotic rectal surgery—have been published [55–57]; although different methods and questionnaires have been used by the authors for assessment of this topic, there are some converging evidences in all these studies. A similar trend for sexual function has been observed, with a decrease at one month after surgery and subsequent progressive recovery, and all the authors agree that an easier identification of the nerves and of the planes of dissection is provided by the robotic system (Table 5).

Further research is needed to provide definitive scientific evidence that enhanced precision given by the robotic system permits better preservation of the hypogastric plexus and therefore better functional results. We should also fill the gap in our knowledge about sexual function and quality of life in women treated for rectal cancer, as the vast majority of the trials for both open and minimally invasive surgery assessed male patients only. To the best of the authors’ knowledge, our study represents the largest series ever published and the only evaluating also female sexual function [57].

Oncologic safety has been definitely demonstrated for laparoscopy approach, with some concern about the CRM involvement, as reported in CLASICC trial [14]; the robotic series so far available demonstrate comparable oncologic results with laparoscopy. In [29], the number of lymph nodes retrieved was superior in robotic group, but percentage of involvement of CRM and radial margins were the same as laparoscopy (Table 6). In terms of oncologic safety, open rectal resection is still considered the standard surgical approach. In [45], 200 patients were prospectively evaluated (100 open and 100 robotic), the mean distal resection margin was significantly longer in the robotic than in the open group (2.7 versus 1.9 cm, *P* = 0.001). Moreover, multivariate analysis that included potent parameters, i.e. SSO types, preoperative CRT, T-category, and growth pattern, showed that patients with distal resection margin ≥2 cm were significantly more likely to be in the robotic than in the open group (OR, 2.415; 95% CI, 1.233–4.73; *P* = 0.01). Positive CRMs were observed in one patient from each of the two groups. The mean number of harvested lymph nodes and lymphovascular or perineural invasion was similar in the two groups. In the retrospective analysis of de Souza [46], there were not significant differences in distal and circumferential margin involvement, but in robotic group none CRM was involved and in open group, three out of 46 patients had a positive CRM (Table 7). In our early experience of first 25 robotic rectal resections compared with 25 laparoscopic rectal resections there was one (4%) positive CRM in the laparoscopic group and none in the robotic group, a difference not statistically significant. The number of harvested lymph nodes was slightly superior in the robotic (18) than in laparoscopic group (17), but again the difference was not statistically significant [21]. In all the meta-analyses comparing laparoscopic with robotic rectal resection, a significant difference in the quality of oncologic resection failed to be demonstrated [39–43]. In summary, oncologic safety of robotic rectal resection was clearly demonstrated in term of proximal, distal and circumferential margin clearance, and number of retrieved lymph nodes. No differences in CRM involvement were detected in comparison with open series, and concern expressed in the CLASICC trial for CRM involvement of laparoscopy-treated cases is not confirmed in the available robotic series.

## Costs of robotic surgery

Robotic surgery is expensive, due to the cost of robot purchase, maintenance, and instruments. Therefore, a careful analysis of costs and of cost/effectiveness is mandatory, even if it is difficult to perform because of the lack of available data about the potential benefits of robotic surgery. A recent costs analysis in [58] demonstrates that robotic surgery is more expensive with a significantly lower hospital profit in a robotic group compared with a laparoscopic group. The authors underlined the necessity to demonstrate with controlled randomised trials potential oncological and clinical benefits of robotic TME to justify its costs.

On the contrary, the interesting study [59] demonstrates that robotic gastric bypass is less expensive than the equivalent laparoscopic and open operations. The economic advantage is correlated to the lower number of leakages and consequent increasing of the hospital stay costs, and to the absence of mechanical staplers use in the robotic subgroup. It is unlikely that in rectal surgery mechanical staplers will be abandoned, but the possibility to perform a safe robotic rectal suture—associated to the transanal removal of the specimen—is already demonstrated, as described in [60].

The costs/analysis in [61] on robotic prostatectomy summarises accurately the current status of the debate on costs of robotic surgery. The authors underline that robotic surgery provides similar postoperative outcomes of laparoscopic surgery but a reduced learning curve, although costs are currently high, increased competition from manufacturers, and wider dissemination of the technology could drive down costs.

Turchetti *et al* [62] performed a review of the literature on the costs of robotic surgery. They conclude that all the evaluated studies performed a pure costs/analysis, without an evaluation of the effectiveness of robotic surgery. The higher costs of robotic surgery are mainly due to the high purchase and maintenance costs for the robot and to a lesser extent to the longer operating room time. However, emerging evidence shows that operating room time decreases with experience in the use of the robot. Turchetti *et al* overlooked two aspects of robotic surgery, the possibility to facilitate some difficult minimally invasive procedures and the changing role of the assistant at the operating table. Concerning the possibility to increase the number of surgeons able to perform advanced minimally invasive procedures, the authors conclude that if this will be demonstrated by prospective studies the high initial investment in robotic technology may be more than justified.

One of the key steps to pursue a successful robotic programme is the surgical volume. It is strictly connected to the learning curve and to the quality of outcomes. Three to five cases per week during the initiation of the programme are necessary to obtain continuity in the learning curve. Palmer *et al* [63] reported a significant increase in surgical volume since the introduction of the robotic programme, from 40 to 350 cases per year within five years. The cost of surgery can be evaluated with an analysis of the variable costs and the fixed costs. Variable costs are related to all those activities that are necessary to produce the surgical performance (such as disposable tools, medications, and so on). Fixed costs are represented by the overall operating room time dedicated to robotics and the purchase of the system. It is clear that a high surgical volume centre can have an impact in terms of variable costs reduction; hence, the best chance to increase surgical volume and therefore to reduce costs is to share the use of the robotic system with all surgical teams, including urologists, gynaecologists, thoracic surgeons, and other specialties [64].

## Conclusions

Minimally invasive surgery for rectal cancer is still confined to only a few centres, despite the evidence of better short-term clinical outcomes and at least the same oncologic safety of traditional open surgery. The reason for this low diffusion is mainly technical, and robotic surgery seems to overcome some of the limitations of laparoscopy, offering an easy technique with less fatigue for the surgeons and a better possibility of teaching for the beginners. The main concern is related to the high costs of robotic surgery, which can be sustained by a careful economic plan and by the use of the robot spread between different surgical specialties. Furthermore, if it will be demonstrated that an easier technique such as robotics may increase the number of minimally invasive rectal resections, the following costs/effectiveness analysis probably will be in favour of robotic surgery. The results of the ROLARR randomised controlled clinical trial are awaited to assess potential clinical and oncological benefits of robotic surgery in rectal cancer, which can justify its higher costs, as well as an enlargement of competition between the companies producing robots, that could reduce the costs of instrumentation.

## Figures and Tables

**Table 1.** Main differences in results between CLASICC and COREAN trials.

	CLASICC [14], 2005	COREAN [15], 2010
Conversion rate	82/242 (34%)	2/170 (1.2%)
Thirty-day morbidity in converted patients	48/82 (59%)	NA
CRM involvement	30/193 (16%)	5/170 (2.9%)
Mean procedures per surgeon (*N*)	20	75
Participating centres (*N*)	27	3
Mean BMI (kg/m^2^)	25	24.1

NA: not assessed.

CRM: circumferential resection margin.

BMI: body mass index.

**Table 2.** Conversion rate in robotic and laparoscopic surgery for rectal cancer.

	Conversion rate		*p* value
	ROB	LAP	
Park [29], 2010	0/41 (0%)	0/82 (0%)	*1.0*
Kim [25], 2010	2/100 (2%)	3/100 (3%)	*1.0*
Kwak [32], 2011	0/59 (0%)	2/59 (3.4%)	*0.496*
Baek [31], 2011	3/41 (7.3%)	9/41 (22%)	*0.116*
Bianchi [21], 2010	0/25 (0%)	1/25 (4%)	*1.0*
Baik [28], 2009	0/56 (0%)	6/57 (10.5%)	*0.013*
Patriti [35], 2009	0/29 (0%)	7/37 (19%)	*<0.05*

ROB: robotic procedure.

LAP: laparoscopic procedure.

**Table 3.** Personal series: results of robotic resection for distal rectal cancer.

Number of patients (01/2007–12/2012)	259
Mean operating time	240 min (170–420)
Conversion rate	6/259 (2.3%)
Anastomotic leakages	20/259 (7.7%) (Four reintervention)

**Table 4.** Short-term clinical outcomes of robotic and laparoscopic surgery for rectal cancer.

	Hospital stay (days)			Post-op complications (%)		
	ROB	LAP	*p*	ROB	LAP	*p*
Park [29], 2010	9.9	9.4	*0.5*	29.3	23.2	*0.4*
Kim [25], 2010	11.7	14.4	*0.006*	20	27	*0.4*
Kwak [32], 2011	na	na		32	27	*ns*
Baek [31], 2011	6.5	6.6	*0.8*	22	27	*1*
Bianchi [20], 2010	6.5	6	*0.4*	16	24	*0.5*
Baik [28], 2009	5.7	7.6	*0.001*	10.7	19.3	*0.025*
Patriti [35], 2009	11.9	9.6	>*0.05*	30.6	18.9	>*0.05*

na: not assessed.

ns: not significant.

ROB: robotic procedure.

LAP: laparoscopic procedure.

**Table 5.** Urinary and sexual dysfunctions results in robotic rectal resection.

Reference	Study	Results
Kim [55], 2012	39 LAP versus 30 ROB (urinary)	Earlier recovery of normal voiding and sexual function
	20 LAP versus 18 ROB (sexual *male only*)	
D’Annibale [56], 2013	30 LAP versus 30 ROB (*male only*)	Erectile function was restored completely in the ROB group and partially in the LAP group
Luca [57], 2013	74 ROB (38 *males* and 36 *females*)	Sexual function and general sexual satisfaction were restored completely. Urinary function unchanged after surgery

LAP: laparoscopic rectal surgery.

ROB: robotic rectal surgery.

**Table 6.** Oncologic results of robotic and laparoscopic surgery for rectal cancer.

	LNs (mean *N*)			Distal margin (mean, cm)			Positive CRM (%)		
	ROB	LAP	*p*	ROB	LAP	*p*	ROB	LAP	*p*
Park [29], 2010	17.3	14.2	*0.06*	2.1	2.3	*ns*	4.9	3.7	*0.5*
Kim [25], 2010	14.7	16.6	*ns*	2.7	2.6	*0.09*	3	2	*ns*
Kwak [32], 2011	20	21	*0.7*	2.2	2.0	*0.8*	1.7	0	*>0.9*
Baek [31], 2011	13	16	*0.07*	3.6	3.8	*0.6*	2.4	4.9	*1*
Bianchi [21], 2010	18	17	*0.7*	2	2	*1.0*	0	4	*0.9*
Baik [28], 2009	18.4	18.7	*0.8*	4	3.6	*0.4*	7	8	*0.7*
Patriti [35], 2009	10.3	11.2	*>0.05*	2.1	4.5	*>0.05*	0	0	*ns*

LNs: lymph nodes.

CRM: circumferential resection margin.

ns: not significant.

ROB: robotic procedure.

LAP: laparoscopic procedure.

**Table 7.** Oncologic results of open and robotic surgery for rectal cancer.

	LNs (mean)			Distal margin (mean, cm)			Positive CRM (%)		
	ROB	OPEN	*p*	ROB	OPEN	*p*	ROB	OPEN	*p*
De Souza [46], 2011	15	16.8	0.26	na	na		0	3	0.25
Kim [45], 2012	20	19.6	0.7	2.7	1.9	0.001	1	1	1
Park [44], 2011	19.4	18.5	0.06	2.8	2.3	0.002	1	2	0.9

LN: lymph nodes.

CRM: circumferential resection margin.

na: not assessed.

ROB: robotic procedure.

OPEN: laparotomic procedure.
